# General practitioners in Styria – who is willing to take part in research projects and why? A survey by the Institute of General Practice and Health Services Research

**DOI:** 10.1007/s00508-017-1244-5

**Published:** 2017-08-09

**Authors:** Stephanie Poggenburg, Manuel Reinisch, Reinhild Höfler, Florian Stigler, Alexander Avian, Andrea Siebenhofer

**Affiliations:** 1 Institute of General Practice and Health Services Research, Medical University of Graz, Auenbruggerplatz 20/III, 8036 Graz, Austria; 2 Institute for Medical Informatics, Statistics and Documentation, Medical University of Graz, Graz, Austria; 3 Institute of General Practice, Goethe University, Frankfurt am Main, Germany

**Keywords:** Health services research, General practice, Practice-based research network, Motivating factor

## Abstract

Increasing recognition of general practice is reflected in the growing number of university institutes devoted to the subject and Health Services Research (HSR) is flourishing as a result. In May 2015 the Institute of General Practice and Evidence-based Health Services Research, Medical University of Graz, initiated a survey of Styrian GPs. The aim of the survey was to determine the willingness to take part in HSR projects, to collect sociodemographic data from GPs who were interested and to identify factors affecting participation in research projects. Of the 1015 GPs who received the questionnaire, 142 (14%) responded and 135 (13%) were included in the analysis. Overall 106 (10%) GPs indicated their willingness to take part in research projects. Factors inhibiting participation were lack of time, administrative workload, and lack of assistance. Overall, 10% of Styrian GPs were willing to participate in research projects. Knowledge about the circumstances under which family doctors are prepared to participate in HSR projects will help in the planning of future projects.

## Background

The provision of primary health care by general practitioners (GPs) is an essential component of the healthcare system, and is rising in importance as a result of the increasing prevalence of chronic diseases and multimorbidity in an aging society [1]. Political attention [2] is increasingly focusing on shoring up primary care, but valid and informed decisions can only be made if relevant research is conducted in family practices. There is a long tradition of practice-relevant health services research (HSR) in many countries, but this is not the case in Germany and Austria [3], where the number of publications by GPs is much smaller than in Great Britain, the United States, Australia, Canada and The Netherlands [4]. Although primary care offers many opportunities for research, far too few studies are concerned with relevant everyday medical problems, and those few are often based on idealized and underrepresented patient groups [5]. The HSR plays an important role in studies of this kind: it is an interdisciplinary approach that uses scientific methodology to study processes, results and the external conditions of healthcare [6]. In the United States, the importance attached to HSR is reflected in the substantial financial support it has received since the 1960s [7, 8]. State support for such research has been available in Great Britain since the 1980s and for more than a decade in Germany, where HSR studies are financed via national funding programs [9, 10]. In Austria in 2013, the little-known subject of HSR was included in the Federal Target Control Contract 2013–2016 [11], but there remains a glaring lack of available funding.

The Austrian health system is characterized by a focus on inpatient care, and the quality of primary care is clearly below the Organization for Economic Cooperation and Development (OECD) average [12]. Unlike other countries, there is no gatekeeping system in Austria which means the flow of patients is unguided, leading to immense costs [13]. As the “specialist of the discipline of general practice” qualification does not exist in Austria and academic general practice has only a short history at medical universities in the country, there is only a tiny number of HSR projects in which GPs are actually involved. Even though many studies (such as those on polypharmacy [14, 15]) have clearly demonstrated the need for HSR research in primary care, both the working methods in general practice and the complexity of a setting with many variables and potential confounders represent a challenge [16]. The implementation of HSR in Austria will therefore require greater personnel resources in research institutions. Amongst other things, this will make it possible to involve GPs in research projects and to establish practice-based research networks (PBRN) such as those in the Netherlands and the UK. The existing research networks that have been set up by several Institutes of General Practice should be standardized and accredited, so that relevant clinical studies can be conducted in family practice in Austria [17]; however, it is only possible to carry out such projects if GPs are prepared to participate. Numerous international studies have investigated the factors that encourage GPs to participate in HSR projects conducted within the structure of PBRNs. Most importantly, it is the research projects themselves and their practical significance, i. e. content factors, that motivate GPs to participate [18–20]. The number of participating doctors can be increased significantly by focusing on clinically relevant research topics [21], gauging the interest of potential participants in the subject matter [22], and allowing them to participate in the research [23]. In addition, a personal relationship between researcher and research practice, and the personal recruitment of practices, appear to have a strong motivating influence on readiness to participate [24–26]. For example, the response rate can be raised significantly, simply by personally addressing a letter asking potential participants to fill in a questionnaire. Financial incentives, on the other hand, seem to be of little value [23, 24]. As the current workload of GPs is already substantial, the additional work required by participation in research projects reduces readiness to participate [24, 27, 28].

The aim of the survey by the Institute of General Practice and Evidence-Based Health Services Research (IAMEV) was to determine the willingness of Styrian GPs to take part in HSR projects, and to collect the sociodemographic data of those GPs who were interested; however, we also aimed to investigate the motivating and inhibiting factors for participation in research, and to study the research subjects proposed by Styrian GPs themselves.

## Material and methods

### Development of the questionnaire

After a selective non-systematic literature search in the Google and Pubmed databases, a preliminary item pool was created by three GPs from our Institute, taking into account pre-existing information and questionnaires from the German-speaking countries Germany [29, 30], Switzerland [31] and South Tyrol (Alto Adige) [32].

A pretest was conducted with five other GPs using a semi-structured interview guideline and techniques taken from cognitive psychology such as think aloud and probing methods, as well as paraphrasing. Our objective was to examine the clarity of the questions, problems interviewees experienced with the questionnaire, interest and attention paid to individual questions, and interest and attention during the course of the entire interview [33]. The aim of the pretest was also to examine whether questions were acceptable to interviewees and relevant to the research question. The reason for piloting the questionnaire in this way, and audio-recording and transcribing the results, was to check content validity, relevance, comprehensibility and conciseness. A separate investigation of reliability was not conducted. After the questionnaire had been revised on the basis of the pilot study, it was ready to be sent to Styrian GPs.

### Inclusion and exclusion criteria

All GPs with a practice in Styria were eligible for inclusion in the study (data were based on lists of physicians provided by the Styrian Medical Association). All questionnaires completed online or returned by post or fax by 31.07.2015 were included. Questionnaires that were only partially filled in, and that were not completed following a request by telephone to do so, were excluded, as were partially filled in anonymized questionnaires. For this study, “partially filled in” was defined as missing one or more pages. A failure to answer individual questions was not considered a reason for exclusion. Questionnaires that were returned twice were only taken into account once.

### Structure and content of the questionnaire

The 3‑part questionnaire included 29 items and 6 text boxes for free-text responses (see Fig. 1), 26 items consisted of dichotomous questions (e. g. yes/no or f/m), while 3 required responses on an ordinal scale.

**Fig. 1:**
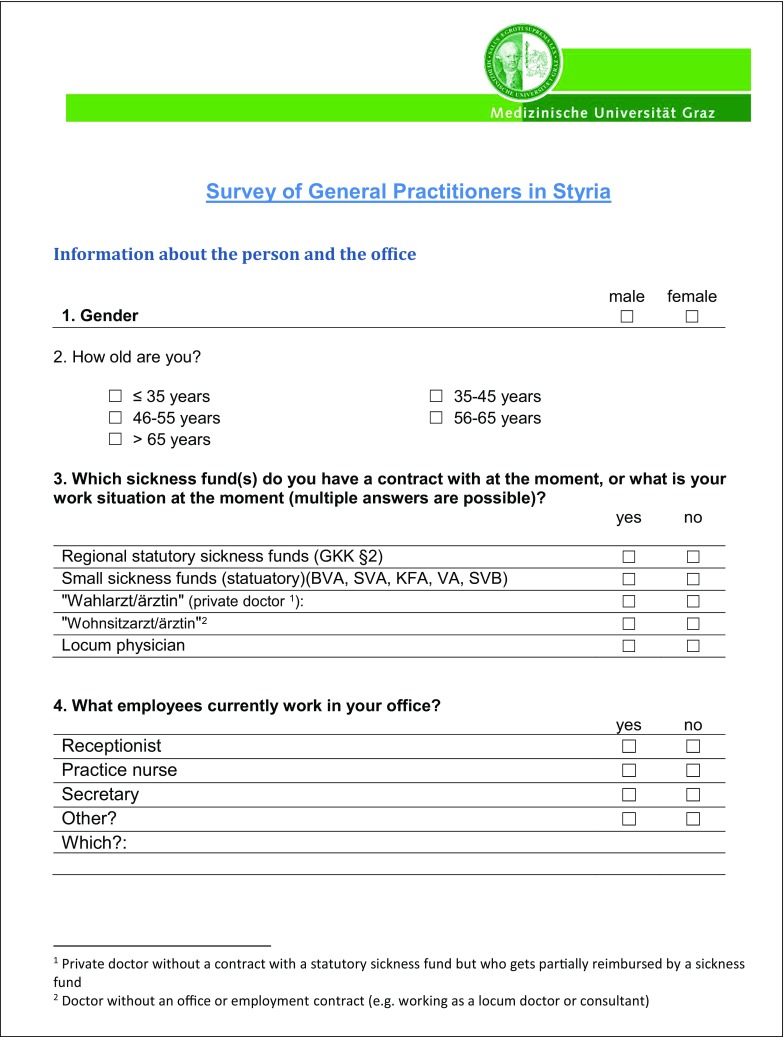
Survey of general practitioners in Styria

**Fig. 1 (continued):**
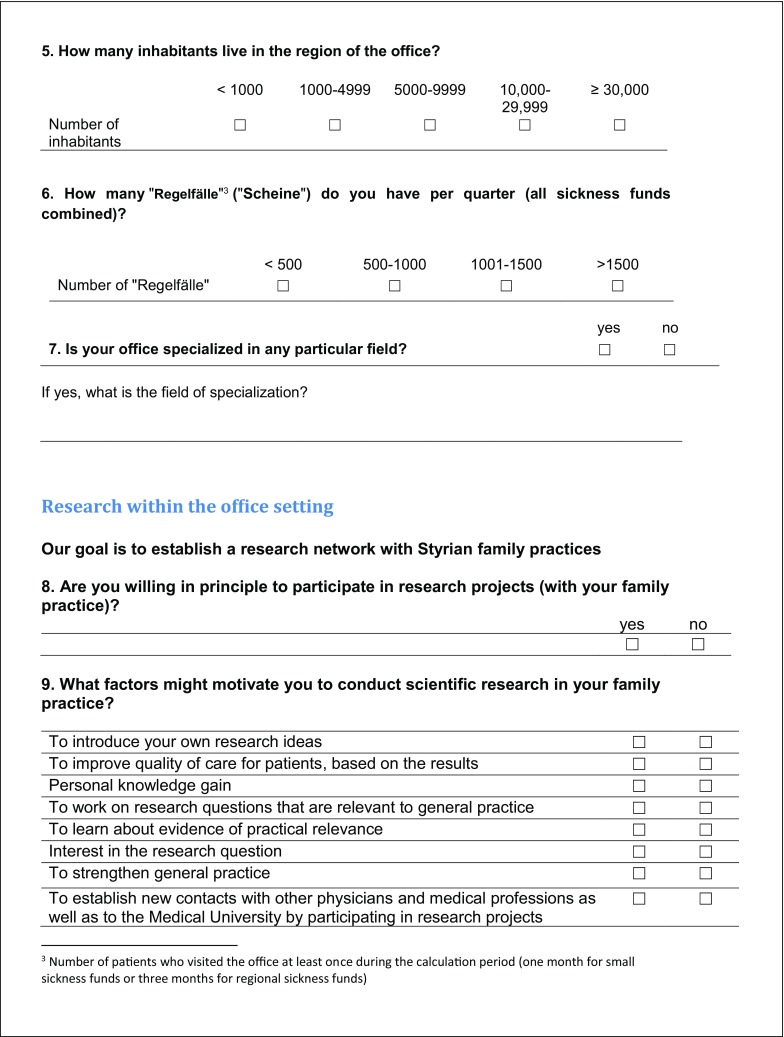
Survey of general practitioners in Styria

**Fig. 1 (continued):**
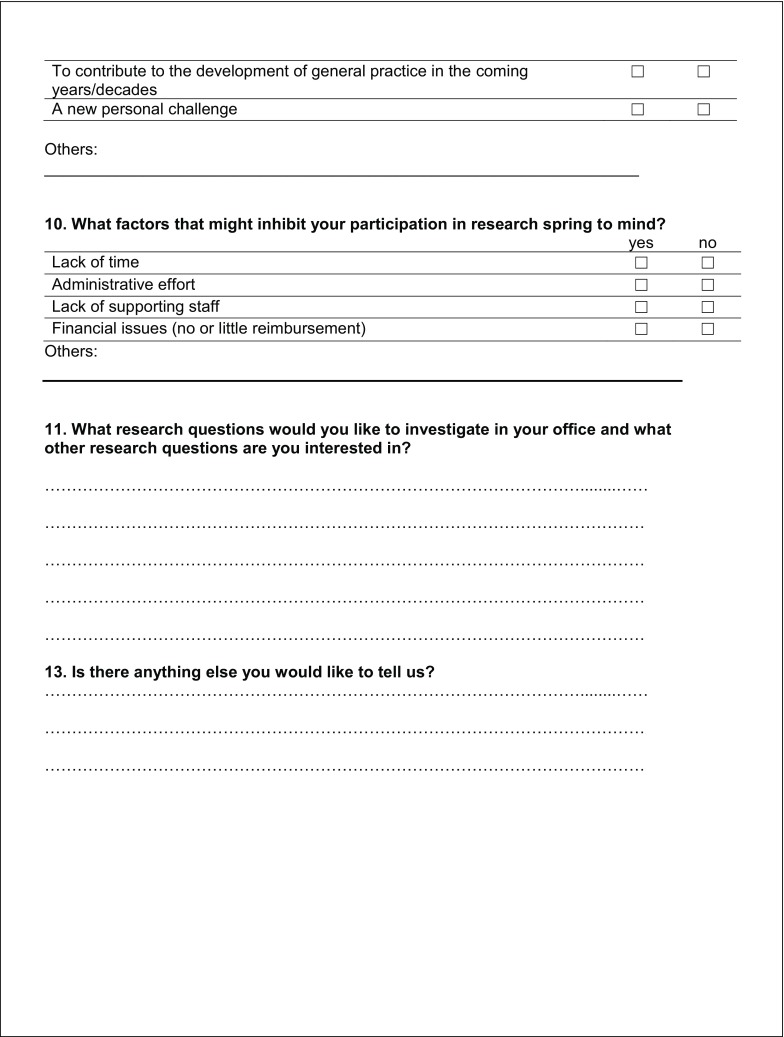
Survey of general practitioners in Styria

**Fig. 1 (continued):**
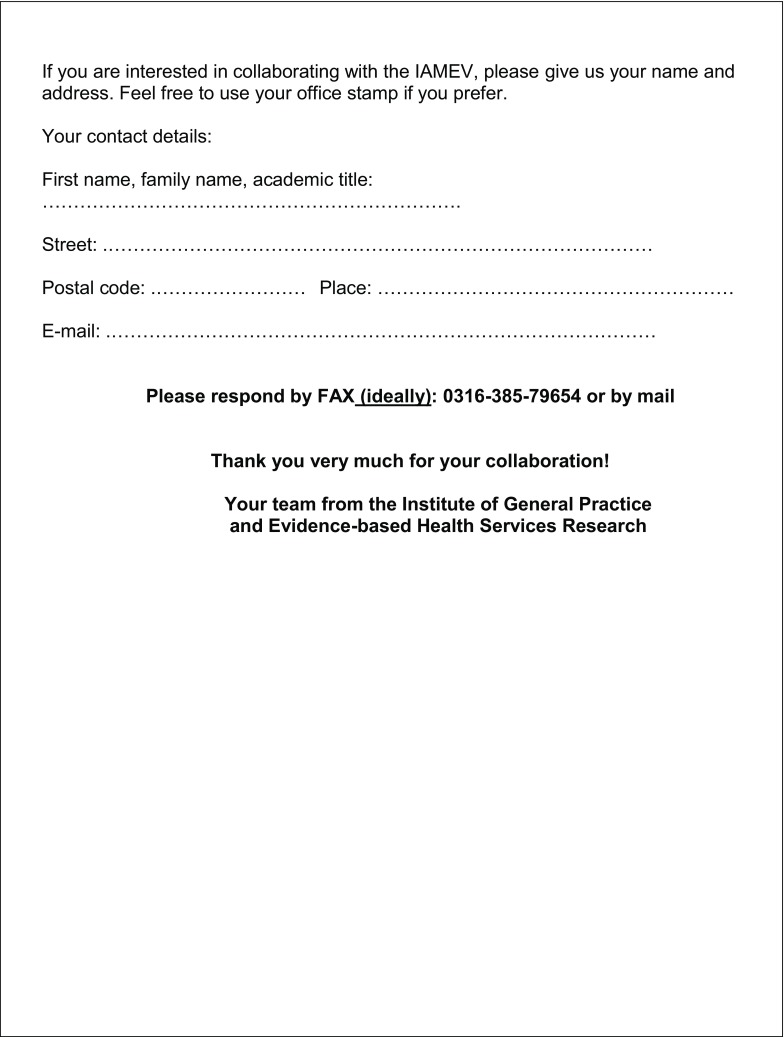
Survey of general practitioners in Styria

The first part asked for sociodemographic data on the physician and the medical practice. The second part consisted of 17 yes/no questions to determine the GP’s interest in research and specifically in taking part in research projects. It also asked about factors motivating and inhibiting participation in scientific research projects. In addition, GPs could add their own research ideas and other comments in the free-text boxes provided. By giving their addresses in the third part of the questionnaire, GPs indicated their willingness to be contacted for future collaboration.

### Addressees

In May 2015 we sent out the questionnaire along with a covering letter from the head of the Institute to all the GPs in Styria (*n* = 1015). It was also possible to answer the survey online via the *umfragen.online.com* website. We sent a reminder to GPs that had not yet responded 2 weeks after the questionnaires were distributed. All responses received by fax, mail or online within 9 weeks of the original mailing were used in the analysis.

### Statistical analysis

Questionnaires with one or more missing pages, duplicates and questionnaires that arrived too late (after 31.07.2015) were excluded from analysis. Data from questionnaires received via mail or fax were entered into a data (excel) sheet. Online responses were exported to an SPSS file and merged with the sheet of data that was compiled offline. SPSS 22 (IBM Corp., Armonk, NY) was used for data analysis. Missing data were not imputed.

We used a qualitative approach to analyze the free-text fields (mainly consisting of research suggestions made by GPs). We collected all answers and carried out inductive categorization. Absolute and relative frequencies of categorical variables (sociodemographic variables, motivational and barrier factors) were provided. A calculation of Cronbach’s alpha was not warranted in the evaluation of our questionnaire because there was the main focus on the GP’s answer to each item rather than the identification of underlying factors.

### Evaluation of the research questions

Research suggestions that GPs entered into the free-text fields were categorized inductively. They were then subsumed under various subject headings. Further evaluation of the research suggestions will be carried out later as part of another research project.

## Results

Of a total of 1015 questionnaires that were sent to Styrian GPs, including 561 males (55.3%) and 454 females (44.7%), 29 were returned to us online, and 113 by mail or fax (total 142), corresponding to a response rate of 14%. After discarding questionnaires that were missing one or more pages, duplicates, and questionnaires that were returned too late, 135 (13%) were included in the final evaluation. Of the responding GPs, 71.1% (*n* = 96) were male and 28.9% (*n* = 39) female. Table 1 shows the sociodemographic and practice-specific parameters.

**Table 1:** Sociodemographic parameters of interviewed general practitioners

Variable				
*Age*	56–65 years: 58 (43%)	46–55 years: 37 (27.4%)	36–45 years: 24 (17.8%)	Under 35 years: 11 (8.1%)
*Contract with sickness fund*	§ 2-Vertrag (regional sickness funds): 86 (78.5%)	Small sickness funds (BVA, SVA, KFA, VA, SVB): 90 (78. 9%)	“Wahlarztstatus” (private doctor^a^): 22 (16%)	“Wohnsitzärzte”^b^: 11 (8.1%)
				Locum physician: 16 (12%)
*Assistance*	Receptionist: 120 (90.2%)	Practice nurse: 34 (25.6%)	Secretary: 48 (36.1%)	*Other employees:* Cleaning worker: 29 Masseur: 7 Physiotherapist: 4 Medical technical assistant: 3
*Number of inhabitants in the area of the office*	<4999: 68 (50.7%)	5000–99,999: 22 (16.4%)	10,000–29,999: 12 (9%)	>30,000: 32 (23.9%)
*“Regelfälle”* ^*c*^ */quarter*	<500 “Regelfälle”: 13 (10.2%)	500–1000 “Regelfälle”: 24 (18.8%)	1001–1500 “Regelfälle”: 61 (47.7%)	>1500 “Regelfälle”: 30 (23.4%)
*Specialized office*	**Yes**: 69 (51.95%)	**No**: 64 (48.1%)	–	–
*Field of practice specialization*	*17 GPs:* Complementary medicine	*8 GPs in each:* Manual medicine Orthopedics Sports medicine	*7 GPs in each:* Nutrition Geriatrics Psychosocial/psychosomatic medicine	*<6 GPs in each:* Preventive medicine Hypertension Pain medicine Occupational medicine Emergency medicine Psychotherapy Wound management
	*15 GPs:* Diabetes mellitus II management			

^a^Private doctor without a contract with the statutory sickness fund who gets partially reimbursed by the sickness fund

^b^Doctor without an office or an employment (e. g. working as a locum doctor or consultant)

^c^Number of patients who visited the office at least once during the calculation period (1 month for small sickness funds or 3 months for regional sickness funds)

At 23.9%, the response rate from urban areas was below average. On the other hand, 47% of GPs from rural areas and particularly from towns with a population of <4999, showed an above-average willingness to take part in research.

### Interest in research

Of the 135 GPs who met the inclusion criteria, 106 (78%) were interested in participating in research projects conducted in a practice setting. The majority of them were male (*n* = 79).

### Motivating factors

The main reasons GPs gave for participating in research projects were upgrading the image of general practice (88.7%), improving quality of care for patients (86.3%), and knowledge gain (86.3%); motivating factors are shown in Table 2.

**Table 2:** Motivating and inhibiting factors for GP participation in research projects

**Motivating factors**	**Percent (%)**
Strengthening general practice	88.7%
Improving quality of care for patients	86.3%
Personal knowledge gain	86.3%
Working on research questions relevant to general practice	77.45%
Learning evidence of practical relevance	76.6%
Contributing to the development of the field of general practice in the coming years/decades	75%
New challenges	56.6%
Introducing own research ideas	43.5%
*Further motivating factors (in the free text field):*	No frequency specification
Encouraging research in alternative- and complementary medicine Quality assurance	
Conveying the importance of general practice to students	
Personal opportunities for cooperation	
**Inhibiting factors**	**Percent (%)**
Lack of time	92.1%
Administrative work involved	81.7%
Lack of support personnel	55.6%
Financial reimbursement	43.7%
*Further inhibiting factors (in the free text field):*	No frequency specification
Age (upcoming retirement/too young and inexperienced)	
Political decision makers and current state of sickness funds make research impossible	

### Inhibiting factors

The main factors inhibiting GPs from participation in general practice research projects were too little time (92.1%), the administrative work involved (81.7%), and lack of assistance (55.6%). All factors, including less important ones such as financial considerations, are shown in Table 2.

### Research questions and subjects

Of the 135 GPs that responded, 66 (48.8%) mentioned a total of 132 research questions or subjects that they considered interesting. The majority of the GPs’ research suggestions concerned questions relating to the role of general practice within the health care system as a whole (*n* = 15), questions involving particular diseases (*n* = 14), questions on complementary medicine and medications in general (*n* = 13), and questions concerning diagnosis and treatment in general practice (*n* = 12). Research suggestions relating to pain, geriatric medicine, organizational issues, quality of care, economics, compliance, health determinants, doctor-patient communication, psychosomatics, nutrition, pregnancy, gender medicine and prevention were all were mentioned 7 times or less.

## Discussion

In our questionnaire, 10% of all Styrian GPs indicated their willingness to take part in research projects, whereby 46.7% of them were older than 55 years. According to Statistik Austria [34], 52% of all physicians in Styria are older than 55. This highlights a major problem with regard to sustainability, since new GPs would have to be recruited regularly, to replace retiring physicians.

Although the proportion of women with their own practices has increased from 15.8% in 1988 to 43.3% in 2015, two thirds of the 10.4% of GPs interested in participating in research were male. The relatively weak interest in answering the questionnaire (returned by only 7.5% of women, as compared to 17.1% of men), combined with even less interest in cooperating on research projects, has already been identified in Germany [30]. Although the proportion of women among both first and senior physician authors of original research in the United States has significantly increased in the last four decades, women are still in the minority [35]. One can only hazard a guess as to the reasons for this worldwide phenomenon, but it may reflect the burden women bear in balancing work and family. Since women are increasingly choosing general practice as a specialty [36], further research should be performed in this area.

Contrary to expectations, GPs from rural areas, and especially from towns with <4999 residents, showed above-average willingness, and more willingness than GPs based in urban areas, to take part in research. As the working conditions of urban and rural GPs vary considerably, it is difficult to determine why this should be the case. Working conditions in rural areas vary depending on specific geographic factors and these influence health in general, as well overall health care, the appropriateness of health care, and health behavior. Consequently, the need for health services research in rural areas is substantial and has considerable potential for improvement [37]. Interest in participating in research was much greater among GPs with relatively low and high incomes. This suggests that the former are motivated to do more than their routine medical work, and that the latter, e. g. private consultants, have more time to participate in research.

It is debatable how and to what extent academia supports research and promote continuing medical education. Discussion is ongoing on the advantages, if any, of the new training regulations for general practice [38]. Whatever these advantages may be, the regulations do not consider the need for scientific research. This is different from other countries, where it has been suggested that GPs involved in training students should receive more instruction in research [39]. Furthermore, trainees involved in research projects are interested in taking part in standardized research training programs [40].

A clear relationship was found between GPs’ special professional interests and their research suggestions. This reflected an interest in developing their personal knowledge as a question of professional interest, and is mirrored in the motivating factors. Research projects are likely to result in an improvement in care for patients receiving treatment in general practice. As improvement in the quality of patient care was a central motivating factor, participating GPs would benefit twice. Studies in Germany have shown that the availability of a health care assistant and/or a secretary may increase willingness to take part in research [41–43]. Health care assistants would benefit from greater job satisfaction, and the quality of research could be expected to increase.

### Motivating factors

The main motivating factors were upgrading general practice as a specialty, improvement in patient care, and acquisition of new and useful professional information. In 2004, Rosemann and Szecsenyi [3] reported that the most important incentive for GPs to take part in research projects was expectations of improved patient care and welfare. These results correlate clearly with results from studies in other countries that indicate that financial reasons are less important than factors relating to content [32, 44, 45]. Research projects that impinge on core areas of general practice and bring GPs the benefit of new information are more likely to motivate them to participate [32] than financial compensation [44]. In the planning stage, future research projects should take relevance to general practice and practicability into account, and ensure that patient outcomes are measurable, as suggested by Kottke et al. in 2008 [45]. In 2013 Peters-Klimm et al. [30] also showed that the strongest motivating factors for participation in a research project were practical relevance and potential for learning.

With the establishment of Institutes of General Practice at Austrian medical universities, e. g. at the private university of Salzburg and at the public university of Graz, as well as a Department of General Practice at the Medical University in Vienna, General Practice has become established as an academic subject in research and teaching. In comparison to neighboring countries, however, Austria has only a short history in this area but great development potential. Nevertheless, the new training regulations have not granted general practice the status of a medical specialty. Upgrading general practice would undoubtedly encourage GPs to show more interest in research.

### Inhibiting factors

As reported in various neighboring European countries, the main factors inhibiting participation in research projects are above all time constraints and the administrative work involved [21, 46–49], along with a lack of administrative personnel and an ambivalent or negative attitude toward research [46, 47, 50].

Although mentioned by 43.7% of respondents, a lack of financial compensation was a relatively unimportant factor in our survey. Results from published studies on financial incentives vary [46, 51]. Nonetheless, a few GPs did mention the need for adequate compensation in the free-text fields of our survey.

It is interesting that no differentiation is made in the literature between “honorarium” and “compensation for expenses” [30]. This suggests that a study design that made few demands on resources and provided adequate compensation for expenses could reduce unwillingness to take part in research projects [44].

Hummers-Pradier et al. noted that besides lack of time and technical and practice-related obstacles (such as lack of computerization), as well as little or no compensation, the vast majority of GPs have no training for or experience in research [52], and would therefore require support. As GPs appear to be willing to take part in such courses, this could take the form of training courses, perhaps provided by the entities that intend to carry out healthcare research studies [30]. It would be possible for a country as small as Austria to follow the example of the UK and set up a nationwide network of research practices [53]. This would simplify the acquisition of valid data and the make it easier to conduct healthcare research projects.

### Strengths and weaknesses

We cannot draw any firm conclusions on the research interest of GPs in Styria since only 10% of GPs responded to our survey. In addition, our results should not be extrapolated to include the whole of Austria, as it was only performed in Styria. Furthermore, it cannot be ruled out that GPs who responded had particular research interests and/or were highly motivated; this would produce a systematic error with respect to the great research interest seen in the results. As a systematic literature search was not carried out before developing the questionnaire, it is further possible that supporting or restraining factors which might influence participation in research projects were overlooked.

As far as we know, no other comparable survey of GPs in Austria has been carried out, so no data for comparison with other Austrian states are available. Data are, however, available for neighboring countries, whereby it should be borne in mind that medical training and continuing medical education can vary considerably from country to country, as can practical working conditions for GPs. Our study does provide information on the structure and number of research-oriented GPs in Styria and may be extended to cover all of Austria at a later date. Knowing what we now do about the motivating and inhibiting factors for GP participation in research projects, concrete measures can be taken to promote healthcare research projects involving GPs.

### Perspectives

Healthcare systems that have a strong primary healthcare structure [54] have better patient outcomes and lower costs than Austria, where there is considerable room for improvement [55]. The importance of primary care is also reflected in recent research [56] where Kringos et al. could show that there is considerable evidence that primary care contributes through its dimensions to overall health system performance and health. Serious efforts should therefore be made to obtain public funds for care research, in order to increase quality of patient care and optimize the use of resources.

As the strength of primary care research in any country is probably a good indicator of the strength and quality of its primary care [57] we should make an effort to establish an efficient PBRN in Styria and Austria as a whole. Now we know more about the factors influencing the willingness of GPs to participate in research projects, it should be easier to motivate them to participate.

## Conclusion

Of the 1015 GPs we wrote to in Styria, 14% responded to our cross-sectional survey, and 10% were interested in participating in health services research projects. They were motivated to do so by an anticipated improvement in patient care and the image of general practice, as well as fresh insights in general. Factors that inhibited them were mainly lack of time and the administrative effort involved, followed by lack of assistance. In the questionnaire, the GPs suggested 132 subjects for research, most of which concerned their own fields of specialization. The willingness of GPs to participate in future HSR projects could be increased by taking the motivating and inhibiting factors into account.
